# Mediterranean Diet, Obesity-Related Metabolic Cardiovascular Disorders, and Environmental Sustainability: A Systematic Review

**DOI:** 10.3390/nu17122005

**Published:** 2025-06-15

**Authors:** Sergio Rodríguez Núñez, María Rubín-García, Vicente Martín-Sánchez, Laura Álvarez-Álvarez, Antonio José Molina

**Affiliations:** 1 Group of Investigation in Interactions Gene-Environment and Health (GIIGAS), Institute of Biomedicine (IBIOMED), Universidad de León, 24007 León, Spain; srodrn@unileon.es (S.R.N.); mrubig@unileon.es (M.R.-G.); vmars@unileon.es (V.M.-S.); ajmolt@unileon.es (A.J.M.); 2 Consortium for Biomedical Research in Epidemiology & Public Health (CIBER Epidemiología y Salud Pública-CIBERESP), 28029 Madrid, Spain

**Keywords:** Mediterranean diet, cardiovascular health, obeso-metabolic syndrome, environmental sustainability

## Abstract

Introduction: This article aims to provide an updated overview of the scientific knowledge regarding the interplay between the Mediterranean diet (MedD), sustainability, and cardiovascular and metabolic health. Methodology: A systematic review was conducted following the PRISMA guidelines, succeeded by a narrative synthesis of data extracted from original research articles in English and Spanish. These articles, indexed in the Scopus and PubMed databases from inception to 31 December 2024, addressed the relationship between MedD, sustainability, and cardiovascular and metabolic health. The methodological quality of the included studies was assessed for bias using the JBI critical appraisal tools. This review was registered in PROSPERO (ID CRD42024476408). Results: The search identified 11 relevant articles. A primary focus on obesity was evident (nine articles), followed by chronic inflammation and metabolic syndrome (two articles each), and cardiovascular health (one article). Regarding sustainability, climate change was the most frequently addressed concern (eight articles). Discussion: A clear trend emerged, indicating a direct association between environmental sustainability, positive health outcomes, and adherence to the MedD. These findings underscore the benefits of the MedD, demonstrating its potential not only to reduce the environmental impact but also to improve health markers such as BMI, metabolic syndrome risk, and chronic inflammation levels.

## 1. Introduction

Increased awareness of climate change has accompanied extreme weather events, such as the Valencia floods in 2024 [1]. Beyond these events, there are broader concerns about this global phenomenon. Mitigating the impact of climate change is critically important for numerous productive sectors, particularly the food system, due to its detrimental effects on soil fertility, crop yields, nutrient composition and bioavailability in food, and crop resistance to pests [2].

Climate change is directly linked to a significant reduction in both food production and food quality [3]. Notably, the food industry is a major contributor to this problem, generating up to one-third of anthropogenic greenhouse gas (GHG) emissions [4].

In this context, different dietary patterns play a crucial role. Shifting away from healthy patterns, such as diets rich in fiber, vegetables, fruits, and cereals [5], towards Western dietary patterns can lead to an increase in GHG emissions [6].

Furthermore, these unhealthy dietary patterns are directly associated with an elevated risk of cardiovascular disease [7], diabetes [8], chronic inflammation [9], overweight or obesity [10], and metabolic syndrome [11]. These dietary shifts have also been linked to an increased risk of other complications, including elevated fasting glucose levels, LDL cholesterol, and hypertension [12].

Addressing the established ‘diet–environment–health trilemma’, food emerges as a central element. Considering these factors, several solutions have been proposed that could positively impact all three components by promoting a shift towards plant-based dietary patterns. Notably, it is estimated that a reduction in meat consumption could lead to an 80% decrease in GHG emissions from food production by 2050 [13].

Among these dietary patterns, the Mediterranean diet (MedD) warrants particular attention. The MedD is a traditional dietary pattern characterized by a high intake of plant-based foods such as fruits, vegetables, legumes, nuts, and whole grains, along with the predominant use of extra virgin olive oil as the main source of fat [14]. It promotes moderate consumption of fish, seafood, and fermented dairy products, while limiting the intake of red and processed meats [15]. Moderate consumption of red wine during meals is optional, and the diet also emphasizes cultural and lifestyle aspects, including home-cooked meals, shared eating practices, and regular physical activity [16].

As a diet primarily based on the consumption of plant-based products, it exhibits a lower carbon footprint [17]. Moreover, it aligns with the four dimensions of sustainability—environmental, social, economic, and nutritional [18]—and has also been demonstrated to offer superior nutritional value compared to other European and American diets [17]. Environmental sustainability, which has been defined as ‘the maintenance of natural capital’ [19], is one of the dimensions studied in this article. It is examined using data such as the carbon footprint.

Furthermore, medical research has consistently demonstrated another significant benefit of the MedD. Studies have indicated its effectiveness in reducing the risk of both primary and secondary occurrences of cardiovascular diseases, type 2 diabetes mellitus, and metabolic syndrome [20,21].

Regarding type 2 diabetes mellitus, dietary interventions such as the MedD have also demonstrated improvements. The MedD involves substantial consumption of fruits, vegetables, and cereals and a moderate intake of white meat and fish, while limiting the intake of red meat, ultra-processed foods, starchy foods, and sugary beverages [22].

Moreover, the Mediterranean region has witnessed a reduction in non-communicable diseases, such as ischemic heart disease and certain types of cancer, alongside an increase in life expectancy [23].

Additionally, the MedD has been shown to support the maintenance of weight loss over time and to reduce the Body Mass Index (BMI), waist circumference, and inflammatory markers such as C-reactive protein (CRP) and low-density lipoprotein (LDL) cholesterol, while increasing high-density lipoprotein (HDL) cholesterol [24]. Consequently, it serves as an effective approach to the management of obesity [25].

Considering all these points, the need to address diet, health, and sustainability as an interconnected whole becomes evident. This interconnectedness is recognized as the diet–health–sustainability trilemma [26].

To date, a systematic review of the trilemma has not been conducted, resulting in a significant knowledge gap that needs to be addressed.

Therefore, this article aims to provide a comprehensive and up-to-date analysis of the scientific evidence regarding the interplay between the MedD, environmental sustainability, and cardiovascular and obesity-related metabolic health.

## 2. Materials and Methods

### 2.1. Design

A systematic review was conducted following the principles of the PRISMA guideline [27] to examine the relationship between adherence to a MedD pattern, environmental sustainability, and health outcomes. The review protocol was registered prospectively in the International Prospective Register of Systematic Reviews (PROSPERO) on 22 March 2024 (ID CRD42024476408). The PRISMA checklist for this article can be found in Table S1.

### 2.2. Search Strategy

A systematic search of the scientific literature was conducted using the Scopus and PubMed databases, encompassing studies from the earliest available records up to 31 December 2024. The search was performed using terms in both Spanish and English, without any restrictions on the age or sex of participants in the studies. To ensure a comprehensive overview, a supplementary search was also conducted in Google Scholar to identify potentially relevant articles that might offer a broader perspective on the topic.

The search strategy centered on three core concepts. Firstly, it focused on the MedD as the primary dietary pattern of interest. Secondly, it addressed environmental sustainability, with a particular emphasis on GHG emissions and the carbon footprint. Thirdly, it encompassed medical aspects, including cardiovascular health, metabolic syndrome, chronic inflammation, diabetes, and obesity, and incorporated relevant indices and parameters such as hypertension and BMI.

To ensure a comprehensive and precise search, a combination of keywords and Medical Subject Headings (MeSH) terms was employed based on predefined criteria. A detailed account of the complete search strategy is presented in Table 1.

### 2.3. Inclusion and Exclusion Criteria

Articles were included in this review if they addressed, at a minimum, the MedD, sustainability, and at least one of the predefined health topics. Furthermore, only studies published from the earliest available date up to 31 December 2024 were considered eligible for analysis.

Studies were excluded if they were review articles, books, book chapters, conference papers, editorials, notes, errata, short questionnaires, or letters. Additionally, articles published in languages other than Spanish or English were not included in this review.

### 2.4. Selection of Studies

Following the removal of duplicate articles and the initial screening based on language and publication type, the remaining articles underwent an independent and blinded review by two researchers (S.R.N. and L.A.-A.). The selection process involved an initial assessment of titles, followed by a review of abstracts. In cases of uncertainty, the full text of the articles was also examined. Subsequently, the selected articles were compiled, and any disagreements in their inclusion were resolved through discussion with a third investigator (A.J.M.).

### 2.5. Extraction of Information

For each article included in this review, the extracted data encompassed the title and the name of the first author. Furthermore, the study design was recorded, categorizing studies as cohort studies, case–control studies, prevalence studies, or other relevant designs.

The assessment methods employed in each study were also documented, including the specific tool used to evaluate adherence to the MedD, such as the Mediterranean Diet Score or MEDAS. Likewise, the approach to measuring sustainability was noted, considering indicators such as CO_2_ production, the carbon footprint, or the ecological footprint.

Additionally, each article was classified based on the specific health topic it investigated, such as cardiovascular health, obesity, diabetes, metabolic syndrome, or chronic inflammation. Lastly, a concise summary of the reported relationship between the studied concepts was compiled to provide a synthesis of the findings. This was made by one researcher (S.R.N.)

### 2.6. Bias Assessment

The JBI critical appraisal tools [28] were utilized to evaluate the bias of the publications by means of an evaluator (S.R.N.) and a reviewer (L.A.-A.).

### 2.7. Synthesis Strategy

A narrative synthesis of the findings was conducted, as a meta-analysis was not performed. The synthesized information was organized thematically, following a structured sequence that began with the MedD, proceeded to sustainability, and concluded with health outcomes.

Within the health domain, the articles included were further categorized based on their primary health focus. This sub-classification encompassed five specific areas: cardiovascular health, obesity, chronic inflammation, metabolic syndrome, and diabetes.

## 3. Results

### 3.1. Summary of the Search and Selection Process

**Figure 1:**
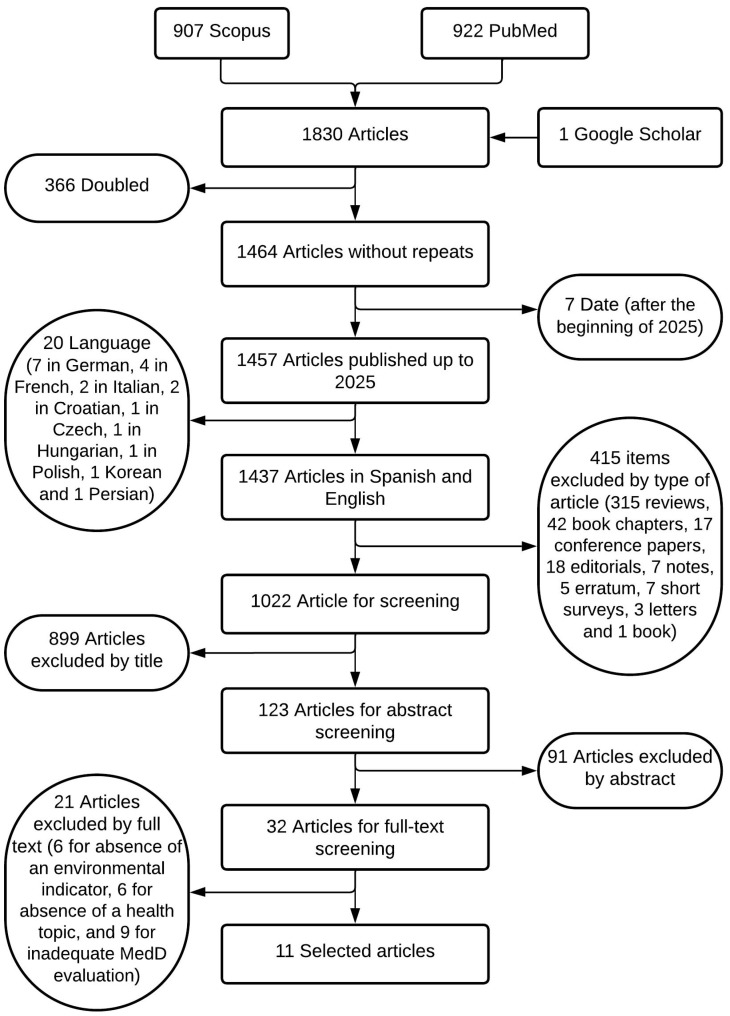
Flow chart of the identification, filtering, and selection of the article-inclusion process as part of the PRISMA 2020 guide.

The article selection process is schematically illustrated in Figure 1. The initial search yielded 1830 articles (907 from Scopus, 922 from PubMed, and 1 from Google Scholar). Following the removal of 366 duplicates, 7 articles were excluded due to publication date (after 31 December 2024), 20 due to language (7 in German, 4 in French, 2 in Italian, 2 in Croatian, 1 in Czech, 1 in Hungarian, 1 in Polish, 1 in Korean, and 1 in Persian), and 415 due to article type (315 reviews, 42 book chapters, 17 conference papers, 18 editorials, 7 notes, 5 errata, 7 short surveys, 3 letters, and 1 book). This process resulted in a total of 1022 articles proceeding to the next stage of screening.

Following the initial search, the screening process led to the exclusion of 899 articles based on their titles (due to the irrelevance to the study’s subject matter) and a further 91 articles based on their abstracts. Of the remaining articles, 21 were excluded after full-text review for the following reasons: absence of an environmental indicator (*n* = 6), absence of a health topic (*n* = 6), and inadequate MedD evaluation (*n* = 9). This multi-stage selection and exclusion process resulted in a final sample of 11 articles for inclusion in the review.

### 3.2. Characteristics of the Studies

The final selection of 11 studies comprised 6 cross-sectional studies, 4 cohort studies, and 1 randomized controlled trial. The key characteristics of these studies are summarized in Table 2. Furthermore, the bias assessment indicated that all articles met the quality criteria for inclusion, achieving a quality score above 69%. The detailed results of the bias assessment are provided in Table S2. Two articles were assessed together due to the use of the same dataset [29,30]. In addition, a concise overview of the demographic characteristics of the study population is provided in Table 3.

### 3.3. Assessment of MedD Adherence

Adherence to the MedD was evaluated in the reviewed articles using various indices, each with specific adaptations. The most frequently employed tools were the Mediterranean Diet Adherence Screener (MEDAS) [31] and the Mediterranean Diet Score (MDS) [32], from which several modified versions have been developed.

From the MEDAS, the energy-restricted MEDAS (er-MEDAS) was developed to assess adherence within the context of caloric restriction [33]. Similarly, adaptations of the MEDAS have been applied to specific populations, such as the Turkish population in the studies by Kocaadam-Bozkurt and Bozkurt [34] and Pınarlı Falakacılar and Yücecan [35], based on previous modifications by Fatma et al. [36] and Bekar and Goktas [37], respectively. In other cases, the original MEDAS indicator was also used without any modifications [38,39].

The MDS, on the other hand, has served as the basis for several adaptations aimed at incorporating information on new food groups or targeting different populations and age groups. Initially, the MDS modification that included fish, developed by the same author [40], was used in a couple of studies [41,42]. The KIDMED index [43] was specifically developed to assess dietary quality in children and adolescents and was employed in the study by Llanaj and Hanley-Cook [44].

Further modifications of the MDS include the alternative Mediterranean Diet Score (aMED), adapted for the U.S. population [45], which was used in the study by Tan and Shin [46]. Additionally, the traditional Mediterranean Diet Score (tMED) [47], designed for non-Mediterranean countries, was applied in Koelman et al. [48]. Lastly, the food frequency-based Mediterranean Diet Score (fMDS) [49], which tailors the MDS for children based on food frequency intake, was utilized in the study by Maritano et al. [50].

**Table 2:** Synthesis of data from the studies selected for the review.

Article	Type of Study	Mediterranean Diet	Sustainability	Health Subject	Health Index	Article Results	Risk of Bias
Fresán et al., 2019 [41]	Cohort study	MDS	GHG and resources consumption	Obesity	BMI	Negative relationship between BMI and MedD	90.91%
Koelman et al. 2023 [48]	Cohort study	tMED	EAT-Lancet score	Chronic inflammation	Chemerin and PCR	Negative relationship between sustainability and the MedD with inflammation	90.91%
Tan and Shin, 2023 [46]	Cohort study	aMED	GHG	Obesity and MS	BMI and MS	Positive relationship between BMI and GHG and between GHG and MS risk	72.73%
Maritano et al., 2024 [50]	Cohort study	fMDS	GHG and land use	Obesity	BMI	Negative relationship between GHG and land use with MedD	90.90%
Llanaj and Hanley-Cook, 2021 [44]	Cross-sectional study	KIDMED	EAT-Lancet score	Obesity and cardiovascular	BMI and DASH	Negative relationship between BMI and land use with MedD	75.00%
García et al., 2023 [30]	Cross-sectional study	er-MEDAS	GHG	Obesity and MS	BMI and MS	Positive relationship between BMI and MS with GHG	100.00%
Gualtieri et al., 2022 [38]	Cross-sectional study	MEDAS	GHG and water footprint	Obesity	BMI	Positive relationship between BMI and GHG and negative between environmental impact and MedD	85.71%
Kocaadam-Bozkurt and Bozkurt, 2023 [34]	Cross-sectional study	MEDAS	SHE	Obesity	BMI	Positive relationship between BMI and GHG	75.00%
Pınarlı Falakacılar and Yücecan, 2024 [35]	Cross-sectional study	MEDAS	GHG and water footprint	Obesity	BMI	Negative relationship between GHG and water footprint with MedD	75.00%
Teixeira et al., 2024 [42]	Cross-sectional study	MDS	GHG and eutrophication	Obesity	BMI	No relationships where found between environmental impact, obesity and MedD	75.00%
Monserrat-Mesquida et al., 2023 [39]	Randomized controlled trial	MEDAS	GHG	Chronic inflammation	DII	Negative relationship between inflammation with GHG and MedD	69.23%

MDS: Mediterranean diet score; tMED: traditional Mediterranean diet score; aMED: alternative Mediterranean diet score; fMDS: food frequency-based Mediterranean diet score; KIDMED: Mediterranean diet quality index for children and adolescents; MEDAS: Mediterranean diet adherence screener; er-MEDAS: energy restriction in the Mediterranean diet; GHG: greenhouse gas emissions; SHE: Sustainable and Healthy Eating Index; BMI: Body Mass Index; PCR C-reactive protein; MS: metabolic syndrome; DASH: Dietary Approaches to Stop Hypertension; DII: Dietary Inflammatory Index; MedD: Mediterranean diet.

**Table 3:** Overview of population of study.

First Author	Year	Nº Patients	Mean Age (years)	% Women	Country
Fresán. Ujué [41]	2019	18,429	36.50	61.79%	Spain
Koelman L. [48]	2023	636	50.8	47.64%	Germany
Tan. Li-Juan [46]	2023	41,659	54.4	69.16%	Republic of Korea
Maritano S. [50]	2024	3358	4.5	49.60%	Italy
Llanaj. Erand [44]	2021	289	18–24	0.87	Albania
García. Silvia [30]	2023	6646	32.5	48.42%	Spain
Gualtieri. Paola [38]	2023	3353	36.00	76.10%	Italy
Kocaadam-Bozkurt. Betül [34]	2023	1333	24.2	61.80%	Turkey
Pınarlı Falakacılar Ç [35]	2024	160	21.13	85.60%	Turkey
Teixeira B. [42]	2024	521; 632	5.7; 13.3	49.3%; 48.7%	Portugal
Monserrat-Mesquida. Margalida [39]	2023	100	55–75	Not specified	Spain

### 3.4. Assessment of Environmental Sustainability

Dietary environmental sustainability was evaluated in the selected studies using a range of methodologies. Several studies assessed sustainability through established food sustainability indices, including those used by Llanaj & Hanley-Cook [44] and Koelman et al. [48]. Knuppel et al. [51] employed the EAT-Lancet score, which aligns with the principles established by the EAT-Lancet Commission on Food, Planet, and Health. Meanwhile, Kocaadam-Bozkurt & Bozkurt [34] utilized the Sustainable and Healthy Eating Index (SHE), adapted for the Turkish population [52].

The majority of the remaining studies focused on carbon footprint analysis. García et al. [30] and Monserrat-Mesquida et al. [39] estimated the carbon footprint using data from the 2016 European database. In contrast, Tan & Shin [46] developed a custom database for the Korean population based on the criteria of the International Standardization Organization guidelines. Gualtieri et al. [38] utilized an online public resource from the World-Wide Fund for Nature (WWF) to assess the carbon footprint and additionally examined the water footprint. Similarly, Pınarlı Falakacılar & Yücecan [35] drew upon various Turkish studies to evaluate both the carbon and water footprints.

Two studies, Maritano et al. [50] and Teixeira et al. [42], utilized the SHARP-Indicators database to assess the carbon footprint and also included land use as an environmental indicator. Finally, Fresán et al. [41] broadened the sustainability analysis by evaluating the carbon footprint together with soil, water, and energy consumption, following a methodology previously detailed by the same author in an earlier publication.

### 3.5. Health Themes

The health themes analyzed in the selected articles were categorized into five groups: cardiovascular health, obesity, chronic inflammation, metabolic syndrome, and diabetes.

Among the eleven articles included in this review, one [44] incorporated cardiovascular health parameters into its investigation. This study evaluated diet using the Dietary Approaches to Stop Hypertension (DASH) index, as described in the study by Mellen et al. [53].

Obesity is a frequently addressed health concern among the articles examined in this review. Body Mass Index (BMI) was a commonly reported measure, appearing in the majority of the studies, with only two exceptions [39,48].

Two of the selected articles focused on chronic inflammation. One study [48] assessed the levels of chemerin and high-sensitivity C-reactive protein (hs-CRP), while the other [39] utilized the Dietary Inflammatory Index (DII).

Two articles within this review addressed metabolic syndrome [30,46]. Both studies assessed metabolic syndrome using the odds ratio for the five diagnostic criteria: elevated triglyceride levels, elevated fasting plasma glucose levels, high blood pressure, low high-density lipoprotein (HDL) levels, and high waist circumference. The presence of three or more of these criteria was required for a diagnosis of metabolic syndrome. Finally, the screening process did not identify any articles that specifically studied diabetes and met the inclusion criteria for this review.

### 3.6. Article Results

A positive association was observed between eating at home and eating in restaurants, with higher adherence to both the MDS (β = −0.38, *p* < 0.001) and the DASH diet (β = −0.23, *p* = 0.003) reported for those who ate at home more frequently [44].

The association between Body Mass Index (BMI) and adherence to the MedD was frequently investigated in the reviewed studies. The majority of findings indicated a negative correlation, suggesting that higher adherence to the MedD was generally associated with a normal BMI with an R^2^ = 0.7 [38], for example. However, this inverse relationship was not statistically significant in some cases [34,41,44], and one study reported no association between these variables (β = −0.096 for children and β = 0.034 for adolescents) [42].

Furthermore, Body Mass Index (BMI) and environmental impact were frequently found to be directly related, with higher BMI values associated with greater CO_2_ production and slightly for water consumption with a Cohen’s d between normal weight and obese of 0.42, for example [30,34,38,46]. However, one study reported no statistically significant association between these factors (R^2^ = 1.6% for children and R^2^ = 0.1% for teenagers) [44].

Moreover, adherence to the MedD showed a negative correlation with environmental impact, indicating that greater adherence was associated with lower greenhouse gas (GHG) (R^2^ = 0.97, as an example) emissions and resource consumption [35,38,50].

Although the MedD was generally presented as a sustainable dietary option, one study indicated that a vegetarian diet might have a lower environmental impact [41]. Gender-based differences in adherence were also noted, with men showing higher adherence at the lowest and highest quintiles, while women exhibited lower adherence values corresponding to the second and fifth greenhouse gas (GHG) emission quintiles [46].

Concerning chronic inflammation, studies observed that lower sustainability index scores were associated with higher levels of chemerin and C-reactive protein (CRP), an inflammatory marker. Similarly, lower adherence to the MedD correlated with higher inflammation indicators, although these associations were not statistically significant. This effect was more pronounced in older men with normal weight at the study’s onset. Furthermore, Body Mass Index (BMI) showed a strong positive correlation with chemerin concentration and inflammation [48]. Additionally, another study found an association between higher CO_2_ emissions and lower Dietary Inflammatory Index (DII) values, with a Cohen’s d of −0.47 between the individuals with more and less of 2.6 kg CO_2_/day, as well as reduced MedD adherence with a Cohen’s d of 0.35 with the same groups [39].

Regarding metabolic syndrome, one study found that the odds of developing the condition decreased in both sexes with diets associated with lower greenhouse gas (GHG) emissions. In women, a decrease in all metabolic syndrome components, except for high-density lipoprotein (HDL) cholesterol, was observed. In men, only HDL cholesterol and fasting plasma glucose levels remained statistically unchanged [46].

Conversely, another study reported that the odds of meeting the criteria for elevated glucose, hypertension, low HDL cholesterol, and high waist circumference increased in the middle quartiles of CO_2_ emissions. In contrast, the odds of meeting the criteria for elevated triglycerides decreased as CO_2_ emissions increased [30]. This study also utilized the Metabolic Syndrome Severity Index [54], which integrates various metabolic indicators. The findings indicated that metabolic syndrome severity increased with higher dietary CO_2_ emissions, while greater adherence to the MedD was associated with lower CO_2_ emissions.

## 4. Discussion

This article aimed to provide a comprehensive and current analysis of the scientific evidence regarding the relationship between the MedD, environmental sustainability, cardiovascular health, and obesity-related metabolic health, aligning with the scope of the recent literature [55].

Firstly, we should bear in mind that 81.82% of the articles included in this study focus on obesity and being overweight, while only 18.18% focus on metabolic syndrome and chronic inflammation, and just 9.09% focus on cardiovascular health.

A further consideration is the utilization of a variety of dietary indices in the assessment of the MedD, which has the potential to compromise the validity of the results and the reliability of the comparisons. Even so, there is a study that compares the MedD indexes and finds a high correlation between some of them, such as the MDS and aMED. These are the indexes used in this study [56].

With a few exceptions discussed below, a concurrent and synergistic protective effect on both health and the environment was generally observed in the reviewed studies when the MedD was followed.

This observation is consistent with previous literature reviews, which have suggested that the MedD may act as a protective factor against obesity [57], cardiovascular disease [58], metabolic syndrome [59], and chronic inflammation [60], in addition to its beneficial impact on the environment [61].

The existing literature suggests that the consumption of the MedD may contribute to a later onset of cardiovascular diseases and certain cancers [41], indicating a positive effect in both disease prevention and management. Indeed, our review found that in studies involving obese patients and those with metabolic syndrome, adherence to the MedD was associated with improvements in their health status [39].

Furthermore, specific food components commonly included in the MedD, such as coffee and cereals, have been associated with positive or protective effects against certain diseases, as observed in the context of diabetes [22].

Beyond the health outcomes explored in our review, the existing literature also highlights the potential of the MedD as a protective factor or even a therapeutic approach for other non-communicable diseases, such as certain cancers and neurodegenerative conditions like Alzheimer’s disease, Parkinson’s disease, or dementia [62], as well as non-alcoholic fatty liver disease [63]. Furthermore, the MedD has been associated with other benefits, including healthier aging [64] and improved overall diet quality [55].

Nevertheless, some studies have indicated that the expected positive relationship between MedD adherence and health outcomes is not always observed [41,42]. For instance, these studies found no significant association between BMI and MedD adherence or sustainability, a finding that contrasts with the conclusions of other reviews that evaluated these relationships independently [65,66].

Another noteworthy finding within the obesity and overweight group comes from the study by Llanaj and Hanley-Cook [44], which observed a protective association against dietary adiposity when assessed using the EAT index, but a detrimental association when assessed using the MDS. However, given the relatively small sample size of this study (289 participants), the results should be interpreted with caution and are not definitive.

Furthermore, it is important to consider that various lifestyle factors beyond diet can influence both health outcomes and environmental impact, as demonstrated in the study by Tan and Shin [46]. This research observed that the population group with lower greenhouse gas (GHG) emissions tended to be older, with a higher intake of carbohydrates, greater physical activity levels, lower rates of tobacco and alcohol consumption, a lower level of educational attainment, and a lower overall caloric intake compared to the group with higher emissions.

Although no studies focusing specifically on diabetes met the inclusion criteria for this review, other literature reviews have suggested a protective role for the MedD against this disease [67]. Furthermore, one of the studies included in our analysis indicated that greater adherence to the MedD was associated with a later onset of diabetes [41]. Given the information discussed, future research could usefully focus on addressing the identified knowledge gaps.

Tan and Shin’s [46] study of the South Korean population reported very low adherence to the MedD. However, it is important to consider the significant cultural and geographical differences between South Korea and the Mediterranean region, which result in radically different customs and food availability. Furthermore, the study used a Mediterranean diet adherence index adapted for the United States, which may not fully capture dietary patterns relevant to South Korea. These factors can play a pivotal role in the impact and outcomes of the Mediterranean diet, as is particularly evident in the South Korean case. This could be a focus for future research.

Additionally, the reasons for the effectiveness of the Mediterranean diet in regions around the Mediterranean Sea are pertinent to consider. The diet is characterized by the consumption of a wide variety of vegetables, incorporating a range of preparation methods. It is also notable for its use of traditional methods of disease prevention and treatment. It is evident that these factors result in favorable health outcomes and enhanced adherence. It is imperative that nutritional policies are devised to facilitate maximizing food consumption in a socially acceptable manner. Nevertheless, studies confirm that the Mediterranean diet can be adopted in non-Mediterranean countries with certain adaptations [68].

While the principles of the Mediterranean diet can be adapted to other regions, it is essential to maintain its fundamental characteristics. These include the abundant use of olive oil, high vegetable consumption, moderate-to-high fish and whole grain intake, and moderate red wine consumption. Conversely, the MedD typically involves minimal-to-no consumption of sugary drinks, red and processed meats, dairy products, and sweets [68].

Concerning the sustainability aspect, the study by Fresán et al. [41] suggested that while the MedD can be considered a sustainable dietary pattern, a vegetarian diet may have a slightly lower overall environmental impact when considering various environmental indicators. However, the MedD may offer nutritional advantages over a strictly vegetarian diet. Importantly, both the MedD and vegetarian diets appear to be more environmentally sustainable and healthier than a typical Western dietary pattern.

Current dietary recommendations often emphasize reducing the consumption of animal products to promote both health and environmental sustainability [69]. Consistent with this, other reviews have reported that vegetarian diets have the lowest equivalent CO_2_ emissions, closely followed by predominantly plant-based diets such as the MedD. In contrast, diets heavily reliant on animal products, such as the Paleo diet, the Keto diet, or the Standard American Diet, exhibit significantly higher CO_2_ emissions [70].

It is important to note that adherence to plant-based diets, including the MedD, has recently declined, trending towards patterns similar to the Western diet [71]. While it was previously hypothesized that the increased cost of MedD-related foods contributed to this shift [72], more recent studies, including those reviewed here, suggest that adherence to the MedD may actually reduce dietary costs [38]. In fact, the MedD is now considered one of the most economically sustainable dietary patterns, outperforming both the Western and vegetarian diets in this regard [41]. To promote adherence to the MedD in a manner that is not only healthy and environmentally sustainable but also economically sustainable, consuming meals at home is recommended over eating out [44].

This review possesses several notable strengths and limitations that warrant consideration when interpreting its findings. A key strength lies in the comprehensive search strategy, which simultaneously addressed the MedD, environmental sustainability, cardiovascular health, and obesity-related metabolic conditions. This integrated approach allowed for a more nuanced and precise analysis compared to studies examining these topics in isolation.

Furthermore, the review followed the PRISMA guidelines, ensuring a rigorous and transparent methodological approach. The risk of bias in the included studies was assessed using the JBI critical appraisal tools, and all articles achieved a quality score exceeding 69%, bolstering the confidence in the synthesized evidence.

Nevertheless, several limitations should be considered. The relatively small number of included articles may limit the generalizability of our conclusions. Additionally, the extrapolation of dietary patterns originating from a specific region like the Mediterranean to culturally diverse populations with significantly different resource availability presents a notable challenge.

Furthermore, while the integrated approach of examining the MedD, sustainability, and health outcomes provided a focused analysis, it may have inherently limited the number of identified studies compared to conducting separate searches for each individual topic. A further limitation is that 81.82% of the articles address obesity and overweight, while only 18.18% address chronic inflammation and metabolic syndrome, and 9.19% address cardiovascular diseases. Furthermore, it is important to note that the utilization of a variety of indexes may be regarded as a constraint.

Despite these limitations, this review offers valuable and timely insights into the complex interplay between dietary patterns, human health, and environmental sustainability.

## 5. Conclusions

Although this review explored potential synergies between the MedD, environmental sustainability, and health outcomes, the overall findings reveal a fragmented and often inconclusive evidence base. While some studies—particularly those conducted within or near the Mediterranean basin—reported statistically significant improvements in cardiovascular health and obesity-related metabolic markers, the majority of studies, especially those conducted in non-Mediterranean regions, failed to demonstrate consistent or statistically significant results.

This lack of coherence raises important questions about the universal applicability and effectiveness of the MedD across diverse cultural and geographic settings. Many of the reviewed interventions lacked standardization, and issues related to cultural adaptation, dietary adherence, and measurement heterogeneity further limit the interpretability and generalizability of the results.

Consequently, the current evidence does not unequivocally support the MedD as a globally transferable model for sustainable and healthy eating. More rigorous, context-sensitive research is urgently needed to clarify its long-term health and environmental impacts outside its region of origin, incorporating not only nutritional and ecological dimensions but also socio-economic and cultural factors.

Finally, while the conceptual alignment of predominantly plant-based diets with public health and environmental goals remains compelling, the practical implementation of such dietary patterns—particularly in settings where food systems and cultural habits differ substantially—requires careful consideration and policy support grounded in local realities.

## Figures and Tables

**Table 1:** Search strategy syntaxis.

Scopus	TITLE-ABS-KEY ((“diet, mediterranean” OR (“diet” AND “mediterranean”) OR “mediterranean diet” OR “diet mediterranean”) AND (“sustain” OR “sustainability” OR “sustainable” OR “sustainably” OR “sustained” OR “sustaining” OR “sustainment” OR “sustains” OR (“environ” OR “environment” OR “environment” OR “environments” OR “environment s” OR “environs”) OR (“climate change” OR (“climate” AND “change”) OR “climate change”) OR (“greenhouse effect” OR (“greenhouse” AND “effect”) OR “greenhouse effect”) OR (“sustainable development” OR (“sustainable” AND “development”) OR “sustainable development”) OR “co2” OR (“carbon footprint” OR (“carbon” AND “footprint”) OR “carbon footprint”) OR (“land basel”[Journal] OR “land”) OR (“fresh water” OR (“fresh” AND “water”) OR “fresh water”) OR (“acidification” OR “acidifications”) OR (“eutrophic” OR “eutrophicated” OR “eutrophication” OR “eutrophication” OR “eutrophications” OR “eutrophics” OR “eutrophized”) OR (“water pollution” OR (“water” AND “pollution”) OR “water pollution”) OR (“air pollution” OR (“air” AND “pollution”) OR “air pollution”) OR (“biodiverse” OR “biodiversities” OR “biodiversity” OR “biodiversity”) OR (“conservation of natural resources” OR (“conservation” AND “natural” AND “resources”) OR “conservation of natural resources” OR “deforestation” OR “deforestations” OR “deforested” OR “deforesting”) OR (“overexploitation” OR “overexploited” OR “overexploiting”) OR “WISH”) AND (“overweight” OR “overweight” OR “overweighted” OR “overweightness” OR “overweights” OR (“obeses” OR “obesity” OR “obesity” OR “obese” OR “obesities” OR “obesity s”) OR (“body mass index” OR (“body” AND “mass” AND “index”) OR “body mass index” OR “BMI”) OR (“weight loss” OR (“weight” AND “loss”) OR “weight loss”) OR (“anti obesity agents” OR “anti obesity agents” OR (“anti obesity” AND “agents”) OR “anti obesity agents” OR (“anti” AND “obesity” AND “agents”) OR “anti obesity agents”) OR (“weight reduction programs” OR (“weight” AND “reduction” AND “programs”) OR “weight reduction programs”) OR (“diet, reducing” OR (“diet” AND “reducing”) OR “reducing diet” OR “diet reducing”) OR (“body weight” OR (“body” AND “weight”) OR “body weight”) OR (“heart disease risk factors” OR (“heart” AND “disease” AND “risk” AND “factors”) OR “heart disease risk factors”) OR (“cardiovascular diseases” OR (“cardiovascular” AND “diseases”) OR “cardiovascular diseases”) OR (“blood pressure” OR (“blood” AND “pressure”) OR “blood pressure” OR “blood pressure determination” OR (“blood” AND “pressure” AND “determination”) OR “blood pressure determination” OR “arterial pressure” OR (“arterial” AND “pressure”) OR “arterial pressure”) OR (“metabolic syndrome” OR (“metabolic” AND “syndrome”) OR “metabolic syndrome”) OR ((“chronic” OR “chronical” OR “chronically” OR “chronicities” OR “chronicity” OR “chronicization” OR “chronics”) AND (“inflammation” OR “inflammation” OR “inflammations” OR “inflammation s”)) OR (“diabetes mellitus” OR (“diabetes” AND “mellitus”) OR “diabetes mellitus”) OR (“glycaemic index” OR “glycemic index” OR (“glycemic” AND “index”) OR “glycemic index” OR (“glucose” OR “glucose” OR “glucoses” OR “glucose s”)) OR “WISH” OR (“glycosylated haemoglobin” OR “glycated hemoglobin” OR (“glycated” AND “hemoglobin”) OR “glycated hemoglobin” OR (“glycosylated” AND “hemoglobin”) OR “glycosylated hemoglobin”)))
PubMed	(“diet, mediterranean”[MeSH Terms] OR (“diet”[All Fields] AND “mediterranean”[All Fields]) OR “mediterranean diet”[All Fields] OR “diet mediterranean”[All Fields]) AND (“sustain”[All Fields] OR “sustainability”[All Fields] OR “sustainable”[All Fields] OR “sustainably”[All Fields] OR “sustained”[All Fields] OR “sustaining”[All Fields] OR “sustainment”[All Fields] OR “sustains”[All Fields] OR (“environ”[All Fields] OR “environment”[MeSH Terms] OR “environment”[All Fields] OR “environments”[All Fields] OR “environment s”[All Fields] OR “environs”[All Fields]) OR (“climate change”[MeSH Terms] OR (“climate”[All Fields] AND “change”[All Fields]) OR “climate change”[All Fields]) OR (“greenhouse effect”[MeSH Terms] OR (“greenhouse”[All Fields] AND “effect”[All Fields]) OR “greenhouse effect”[All Fields]) OR (“sustainable development”[MeSH Terms] OR (“sustainable”[All Fields] AND “development”[All Fields]) OR “sustainable development”[All Fields]) OR “co2”[All Fields] OR (“carbon footprint”[MeSH Terms] OR (“carbon”[All Fields] AND “footprint”[All Fields]) OR “carbon footprint”[All Fields]) OR (“land basel”[Journal] OR “land”[All Fields]) OR (“fresh water”[MeSH Terms] OR (“fresh”[All Fields] AND “water”[All Fields]) OR “fresh water”[All Fields]) OR (“acidification”[All Fields] OR “acidifications”[All Fields]) OR (“eutrophic”[All Fields] OR “eutrophicated”[All Fields] OR “eutrophication”[MeSH Terms] OR “eutrophication”[All Fields] OR “eutrophications”[All Fields] OR “eutrophics”[All Fields] OR “eutrophized”[All Fields]) OR (“water pollution”[MeSH Terms] OR (“water”[All Fields] AND “pollution”[All Fields]) OR “water pollution”[All Fields]) OR (“air pollution”[MeSH Terms] OR (“air”[All Fields] AND “pollution”[All Fields]) OR “air pollution”[All Fields]) OR (“biodiverse”[All Fields] OR “biodiversities”[All Fields] OR “biodiversity”[MeSH Terms] OR “biodiversity”[All Fields]) OR (“conservation of natural resources”[MeSH Terms] OR (“conservation”[All Fields] AND “natural”[All Fields] AND “resources”[All Fields]) OR “conservation of natural resources”[All Fields] OR “deforestation”[All Fields] OR “deforestations”[All Fields] OR “deforested”[All Fields] OR “deforesting”[All Fields]) OR (“overexploitation”[All Fields] OR “overexploited”[All Fields] OR “overexploiting”[All Fields]) OR “WISH”[All Fields]) AND (“overweight”[MeSH Terms] OR “overweight”[All Fields] OR “overweighted”[All Fields] OR “overweightness”[All Fields] OR “overweights”[All Fields] OR (“obeses”[All Fields] OR “obesity”[MeSH Terms] OR “obesity”[All Fields] OR “obese”[All Fields] OR “obesities”[All Fields] OR “obesity s”[All Fields]) OR (“body mass index”[MeSH Terms] OR (“body”[All Fields] AND “mass”[All Fields] AND “index”[All Fields]) OR “body mass index”[All Fields] OR “BMI”[All Fields]) OR (“weight loss”[MeSH Terms] OR (“weight”[All Fields] AND “loss”[All Fields]) OR “weight loss”[All Fields]) OR (“anti obesity agents”[Pharmacological Action] OR “anti obesity agents”[MeSH Terms] OR (“anti obesity”[All Fields] AND “agents”[All Fields]) OR “anti obesity agents”[All Fields] OR (“anti”[All Fields] AND “obesity”[All Fields] AND “agents”[All Fields]) OR “anti obesity agents”[All Fields]) OR (“weight reduction programs”[MeSH Terms] OR (“weight”[All Fields] AND “reduction”[All Fields] AND “programs”[All Fields]) OR “weight reduction programs”[All Fields]) OR (“diet, reducing”[MeSH Terms] OR (“diet”[All Fields] AND “reducing”[All Fields]) OR “reducing diet”[All Fields] OR “diet reducing”[All Fields]) OR (“body weight”[MeSH Terms] OR (“body”[All Fields] AND “weight”[All Fields]) OR “body weight”[All Fields]) OR (“heart disease risk factors”[MeSH Terms] OR (“heart”[All Fields] AND “disease”[All Fields] AND “risk”[All Fields] AND “factors”[All Fields]) OR “heart disease risk factors”[All Fields]) OR (“cardiovascular diseases”[MeSH Terms] OR (“cardiovascular”[All Fields] AND “diseases”[All Fields]) OR “cardiovascular diseases”[All Fields]) OR (“blood pressure”[MeSH Terms] OR (“blood”[All Fields] AND “pressure”[All Fields]) OR “blood pressure”[All Fields] OR “blood pressure determination”[MeSH Terms] OR (“blood”[All Fields] AND “pressure”[All Fields] AND “determination”[All Fields]) OR “blood pressure determination”[All Fields] OR “arterial pressure”[MeSH Terms] OR (“arterial”[All Fields] AND “pressure”[All Fields]) OR “arterial pressure”[All Fields]) OR (“metabolic syndrome”[MeSH Terms] OR (“metabolic”[All Fields] AND “syndrome”[All Fields]) OR “metabolic syndrome”[All Fields]) OR ((“chronic”[All Fields] OR “chronical”[All Fields] OR “chronically”[All Fields] OR “chronicities”[All Fields] OR “chronicity”[All Fields] OR “chronicization”[All Fields] OR “chronics”[All Fields]) AND (“inflammation”[MeSH Terms] OR “inflammation”[All Fields] OR “inflammations”[All Fields] OR “inflammation s”[All Fields])) OR (“diabetes mellitus”[MeSH Terms] OR (“diabetes”[All Fields] AND “mellitus”[All Fields]) OR “diabetes mellitus”[All Fields]) OR (“glycaemic index”[All Fields] OR “glycemic index”[MeSH Terms] OR (“glycemic”[All Fields] AND “index”[All Fields]) OR “glycemic index”[All Fields] OR (“glucose”[MeSH Terms] OR “glucose”[All Fields] OR “glucoses”[All Fields] OR “glucose s”[All Fields])) OR “WISH”[All Fields] OR (“glycosylated haemoglobin”[All Fields] OR “glycated hemoglobin”[MeSH Terms] OR (“glycated”[All Fields] AND “hemoglobin”[All Fields]) OR “glycated hemoglobin”[All Fields] OR (“glycosylated”[All Fields] hemoglobin”[All Fields]))

Search based on Medical Subject Headings (MeSH) terms to encompass a more comprehensive search. WISH: ‘World Index for Sustainability and Health’.

## Data Availability

No new data were created or analyzed in this study. Data sharing is not applicable to this article.

## Supplementary Materials

The following supporting information can be downloaded at https://www.mdpi.com/article/10.3390/nu17122005/s1, Table S1: PRISMA 2020 checklist, Table S2: Bias assessment synthesis. Reference [73] is cited in the supplementary materials.

## References

[1] Ceccarelli G., Branda F., Giovanetti M., Ciccozzi M., Scarpa F. (2024). The Urgent Need for Arbovirus Surveillance and Control Following a Catastrophic Event: The Case of the DANA Flood Event in Valencia. New Microbes New Infect..

[2] Owino V., Kumwenda C., Ekesa B., Parker M.E., Ewoldt L., Roos N., Lee W.T., Tome D. (2022). The Impact of Climate Change on Food Systems, Diet Quality, Nutrition, and Health Outcomes: A Narrative Review. Front. Clim..

[3] United Nations (2023). Informe de los Objetivos de Desarrollo Sostenible. https://unstats.un.org/sdgs/report/2023/The-Sustainable-Development-Goals-Report-2023_Spanish.pdf.

[4] Crippa M., Solazzo E., Guizzardi D., Monforti-Ferrario F., Tubiello F.N., Leip A. (2021). Food Systems Are Responsible for a Third of Global Anthropogenic GHG Emissions. Nat. Food.

[5] Fritsch J., Garces L., Quintero M.A., Pignac-Kobinger J., Santander A.M., Fernández I., Ban Y.J., Kwon D., Phillips M.C., Knight K. (2021). Low-Fat, High-Fiber Diet Reduces Markers of Inflammation and Dysbiosis and Improves Quality of Life in Patients with Ulcerative Colitis. Clin. Gastroenterol. Hepatol..

[6] Paris J.M.G., Falkenberg T., Nöthlings U., Heinzel C., Borgemeister C., Escobar N. (2022). Changing Dietary Patterns Is Necessary to Improve the Sustainability of Western Diets from a One Health Perspective. Sci. Total Environ..

[7] Wang Y.J., Yeh T.L., Shih M.C., Tu Y.K., Chien K.L. (2020). Dietary Sodium Intake and Risk of Cardiovascular Disease: A Systematic Review and Dose-Response Meta-Analysis. Nutrients.

[8] Tilman D., Clark M. (2014). Global Diets Link Environmental Sustainability and Human Health. Nature.

[9] Galland L. (2010). Diet and Inflammation. Nutr. Clin. Pract..

[10] Malik V.S., Hu F.B. (2015). Fructose and Cardiometabolic Health: What the Evidence from Sugar-Sweetened Beverages Tells Us. J. Am. Coll. Cardiol..

[11] Durán Agüero S., Carrasco Piña E., Araya Pérez M. (2012). Food and Diabetes. Nutr. Hosp..

[12] Poti J.M., Braga B., Qin B. (2017). Ultra-Processed Food Intake and Obesity: What Really Matters for Health-Processing or Nutrient Content?. Curr. Obes. Rep..

[13] Willett W., Rockström J., Loken B., Springmann M., Lang T., Vermeulen S., Garnett T., Tilman D., DeClerck F., Wood A. (2019). Food in the Anthropocene: The EAT–Lancet Commission on Healthy Diets from Sustainable Food Systems. Lancet.

[14] Rovira J., Ramirez-Bajo M.J., Bañon-Maneus E., Ventura-Aguiar P., Arias-Guillén M., Romano-Andrioni B., Ojeda R., Revuelta I., García-Calderó H., Barberà J.A. (2024). Mediterranean Diet Pattern: Potential Impact on the Different Altered Pathways Related to Cardiovascular Risk in Advanced Chronic Kidney Disease. Nutrients.

[15] Wirth J., Di Giuseppe R., Boeing H., Weikert C. (2016). A Mediterranean-Style Diet, Its Components and the Risk of Heart Failure: A Prospective Population-Based Study in a Non-Mediterranean Country. Eur. J. Clin. Nutr..

[16] Huhn S., Masouleh S.K., Villringer A., Witte A.V. (2015). Components of a Mediterranean Diet and Their Impact on Cognitive Functions in Aging. Front. Aging Neurosci..

[17] Cambeses-Franco C., González-García S., Feijoo G., Moreira M.T. (2022). Driving Commitment to Sustainable Food Policies within the Framework of American and European Dietary Guidelines. Sci. Total Environ..

[18] Bôto J.M., Rocha A., Miguéis V., Meireles M., Neto B. (2022). Sustainability Dimensions of the Mediterranean Diet: A Systematic Review of the Indicators Used and Its Results. Adv. Nutr..

[19] Morelli J. (2013). Environmental Sustainability: A Definition for Environmental Professionals. J. Environ. Sustain..

[20] Castro-Barquero S., Ruiz-León A.M., Sierra-Pérez M., Estruch R., Casas R. (2020). Dietary Strategies for Metabolic Syndrome: A Comprehensive Review. Nutrients.

[21] Delgado-Lista J., Alcala-Diaz J.F., Torres-Peña J.D., Quintana-Navarro G.M., Fuentes F., Garcia-Rios A., Ortiz-Morales A.M., Gonzalez-Requero A.I., Perez-Caballero A.I., Yubero-Serrano E.M. (2022). Long-Term Secondary Prevention of Cardiovascular Disease with a Mediterranean Diet and a Low-Fat Diet (CORDIOPREV): A Randomised Controlled Trial. Lancet.

[22] Xi P., Liu R.H. (2016). Whole Food Approach for Type 2 Diabetes Prevention. Mol. Nutr. Food Res..

[23] Dominguez L.J., Veronese N., Baiamonte E., Guarrera M., Parisi A., Ruffolo C., Tagliaferri F., Barbagallo M. (2022). Healthy Aging and Dietary Patterns. Nutrients.

[24] Guasch-Ferré M., Willett W.C. (2021). The Mediterranean Diet and Health: A Comprehensive Overview. J. Intern. Med..

[25] Muscogiuri G., Verde L., Sulu C., Katsiki N., Hassapidou M., Frias-Toral E., Cucalón G., Pazderska A., Yumuk V.D., Colao A. (2022). Mediterranean Diet and Obesity-Related Disorders: What Is the Evidence?. Curr. Obes. Rep..

[26] Clark M., Hill J., Tilman D. (2018). The Diet, Health, and Environment Trilemma. Annu. Rev. Environ. Resour..

[27] Yepes-Nuñez J.J., Urrútia G., Romero-García M., Alonso-Fernández S. (2021). Declaración PRISMA 2020: Una Guía Actualizada Para La Publicación de Revisiones Sistemáticas. Rev. Esp. Cardiol..

[28] JBI Critical Appraisal Tools | JBI. https://jbi.global/critical-appraisal-tools.

[29] García S., Bouzas C., Mateos D., Pastor R., Álvarez L., Rubín M., Martínez-González M.Á., Salas-Salvadó J., Corella D., Goday A. (2023). Carbon Dioxide (CO_2_) Emissions and Adherence to Mediterranean Diet in an Adult Population: The Mediterranean Diet Index as a Pollution Level Index. Environ. Health.

[30] García S., Pastor R., Monserrat-Mesquida M., Álvarez-Álvarez L., Rubín-García M., Martínez-González M.Á., Salas-Salvadó J., Corella D., Goday A., Martínez J.A. (2023). Metabolic Syndrome Criteria and Severity and Carbon Dioxide (CO2) Emissions in an Adult Population. Global Health.

[31] Schröder H., Fitó M., Estruch R., Martínez-González M.A., Corella D., Salas-Salvadó J., Lamuela-Raventós R., Ros E., Salaverría I., Fiol M. (2011). A Short Screener Is Valid for Assessing Mediterranean Diet Adherence among Older Spanish Men and Women. J. Nutr..

[32] Trichopoulou A., Kouris-Blazos A., Wahlqvist M.L., Gnardellis C., Lagiou P., Polychronopoulos E., Vassilakou T., Lipworth L., Trichopoulos D. (1995). Diet and Overall Survival in Elderly People. BMJ.

[33] Schröder H., Zomeño M.D., Martínez-González M.A., Salas-Salvadó J., Corella D., Vioque J., Romaguera D., Martínez J.A., Tinahones F.J., Miranda J.L. (2021). Validity of the Energy-Restricted Mediterranean Diet Adherence Screener. Clin. Nutr..

[34] Kocaadam-Bozkurt B., Bozkurt O. (2023). Relationship between Adherence to the Mediterranean Diet, Sustainable and Healthy Eating Behaviors, and Awareness of Reducing the Ecological Footprint. Int. J. Environ. Health Res..

[35] Pınarlı Falakacılar Ç., Yücecan S. (2024). The Impact of Sustainability Courses: Are They Effective in Improving Diet Quality and Anthropometric Indices?. Nutrients.

[36] Pehlivanoğlu E.F.Ö., Balcıoğlu H., Ünlüoğlu İ. (2020). Akdeniz Diyeti Bağlılık Ölçeği’nin Türkçe’ye Uyarlanması Geçerlilik ve Güvenilirliği. Osman. Tıp Derg..

[37] Bekar C., Goktas Z. (2023). Validation of the 14-Item Mediterranean Diet Adherence Screener. Clin. Nutr. ESPEN.

[38] Gualtieri P., Marchetti M., Frank G., Cianci R., Bigioni G., Colica C., Soldati L., Moia A., De Lorenzo A., Di Renzo L. (2022). Exploring the Sustainable Benefits of Adherence to the Mediterranean Diet during the COVID-19 Pandemic in Italy. Nutrients.

[39] Monserrat-Mesquida M., Bouzas C., García S., Quetglas-Llabrés M.M., Mateos D., Ugarriza L., Gómez C., Sureda A., Tur J.A. (2023). Carbon Dioxide (CO_2_) Dietary Emissions Are Related to Oxidative and Inflammatory Status in Adult Population. Nutrients.

[40] Trichopoulou A., Costacou T., Bamia C., Trichopoulos D. (2003). Adherence to a Mediterranean Diet and Survival in a Greek Population. New Engl. J. Med..

[41] Fresán U., Martínez-González M.A., Sabaté J., Bes-Rastrollo M. (2019). Global Sustainability (Health, Environment and Monetary Costs) of Three Dietary Patterns: Results from a Spanish Cohort (the SUN Project). BMJ Open.

[42] Teixeira B., Afonso C., Severo M., Carvalho C., Torres D., Lopes C., Oliveira A. (2024). Exploring Dietary Patterns and Their Association with Environmental Sustainability and Body Mass Index in Children and Adolescents: Insights from the National Food, Nutrition and Physical Activity Survey 2015–2016. Sci. Total Environ..

[43] Serra-Majem L., Ribas L., Ngo J., Ortega R.M., García A., Pérez-Rodrigo C., Aranceta J. (2004). Food, Youth and the Mediterranean Diet in Spain. Development of KIDMED, Mediterranean Diet Quality Index in Children and Adolescents. Public Health Nutr..

[44] Llanaj E., Hanley-Cook G.T. (2021). Adherence to Healthy and Sustainable Diets Is Not Differentiated by Cost, but Rather Source of Foods among Young Adults in Albania. Br. J. Nutr..

[45] Fung T.T., McCullough M.L., Newby P., Manson J.E., Meigs J.B., Rifai N., Willett W.C., Hu F.B. (2005). Diet-Quality Scores and Plasma Concentrations of Markers of Inflammation and Endothelial Dysfunction. Am. J. Clin. Nutr..

[46] Tan L.J., Shin S. (2023). Low Greenhouse Gas Emission Self-Selective Diets and Risk of Metabolic Syndrome in Adults 40 and Older: A Prospective Cohort Study in South Korea. Environ. Health Perspect..

[47] Hoffman R., Gerber M. (2013). Evaluating and Adapting the Mediterranean Diet for Non-Mediterranean Populations: A Critical Appraisal. Nutr. Rev..

[48] Koelman L., Herpich C., Norman K., Jannasch F., Börnhorst C., Schulze M.B., Aleksandrova K. (2023). Adherence to Healthy and Sustainable Dietary Patterns and Long-Term Chronic Inflammation: Data from the EPIC-Potsdam Cohort. J. Nutr. Health Aging.

[49] Tognon G., Hebestreit A., Lanfer A., Moreno L.A., Pala V., Siani A., Tornaritis M., De Henauw S., Veidebaum T., Molnár D. (2014). Mediterranean Diet, Overweight and Body Composition in Children from Eight European Countries: Cross-Sectional and Prospective Results from the IDEFICS Study. Nutr. Metab. Cardiovasc. Dis..

[50] Maritano S., Moirano G., Isaevska E., Pizzi C., Ponzo V., Moccia C., Maule M., Lastrucci V., Alderotti G., Ronfani L. (2024). Examining the Relationship between the Environmental Impact of Diet and Child Growth from a Co-Benefit Perspective. Environ. Res..

[51] Knuppel A., Papier K., Key T.J., Travis R.C. (2019). EAT-Lancet Score and Major Health Outcomes: The EPIC-Oxford Study. Lancet.

[52] Köksal E., Bilici S., Dazlroǧlu M.E.Ç., Gövez N.E. (2023). Validity and Reliability of the Turkish Version of the Sustainable and Healthy Eating Behaviors Scale. Br. J. Nutr..

[53] Mellen P.B., Gao S.K., Vitolins M.Z., Goff D.C. (2008). Deteriorating Dietary Habits Among Adults with Hypertension: DASH Dietary Accordance, NHANES 1988–1994 and 1999–2004. Arch. Intern. Med..

[54] Wiley J.F., Carrington M.J. (2016). A Metabolic Syndrome Severity Score: A Tool to Quantify Cardio-Metabolic Risk Factors. Prev. Med..

[55] Leydon C.L., Leonard U.M., McCarthy S.N., Harrington J.M. (2023). Aligning Environmental Sustainability, Health Outcomes, and Affordability in Diet Quality: A Systematic Review. Adv. Nutr..

[56] Olmedo-Requena R., González-Donquiles C., Dávila-Batista V., Romaguera D., Castelló A., de la Torre A.J.M., Amiano P., Dierssen-Sotos T., Guevara M., Fernández-Tardón G. (2019). Agreement among Mediterranean Diet Pattern Adherence Indexes: MCC-Spain Study. Nutrients.

[57] D’innocenzo S., Biagi C., Lanari M. (2019). Obesity and the Mediterranean Diet: A Review of Evidence of the Role and Sustainability of the Mediterranean Diet. Nutrients.

[58] Widmer R.J., Flammer A.J., Lerman L.O., Lerman A. (2015). The Mediterranean Diet, Its Components, and Cardiovascular Disease. Am. J. Med..

[59] Kiortsis D.N., Simos Y.V. (2013). Mediterranean Diet for the Prevention and Treatment of Metabolic Syndrome. Angiology.

[60] Tsigalou C., Konstantinidis T., Paraschaki A., Stavropoulou E., Voidarou C., Bezirtzoglou E. (2020). Mediterranean Diet as a Tool to Combat Inflammation and Chronic Diseases. An Overview. Biomedicines.

[61] Dernini S., Berry E.M., Serra-Majem L., La Vecchia C., Capone R., Medina F.X., Aranceta-Bartrina J., Belahsen R., Burlingame B., Calabrese G. (2017). Med Diet 4.0: The Mediterranean Diet with Four Sustainable Benefits. Public Health Nutr..

[62] Romagnolo D.F., Selmin O.I. (2017). Mediterranean Diet and Prevention of Chronic Diseases. Nutr. Today.

[63] Zelber-Sagi S., Salomone F., Mlynarsky L. (2017). The Mediterranean Dietary Pattern as the Diet of Choice for Non-Alcoholic Fatty Liver Disease: Evidence and Plausible Mechanisms. Liver Int..

[64] Critselis E., Panagiotakos D. (2020). Adherence to the Mediterranean Diet and Healthy Ageing: Current Evidence, Biological Pathways, and Future Directions. Crit. Rev. Food Sci. Nutr..

[65] Koch C.A., Sharda P., Patel J., Gubbi S., Bansal R., Bartel M.J. (2021). Climate Change and Obesity. Horm. Metab. Res..

[66] López-Gil J.F., García-Hermoso A., Sotos-Prieto M., Cavero-Redondo I., Martínez-Vizcaíno V., Kales S.N. (2023). Mediterranean Diet-Based Interventions to Improve Anthropometric and Obesity Indicators in Children and Adolescents: A Systematic Review with Meta-Analysis of Randomized Controlled Trials. Adv. Nutr..

[67] Martín-Peláez S., Fito M., Castaner O. (2020). Mediterranean Diet Effects on Type 2 Diabetes Prevention, Disease Progression, and Related Mechanisms. A Review. Nutrients.

[68] Martínez-González M.Á., Hershey M.S., Zazpe I., Trichopoulou A. (2017). Transferability of the Mediterranean Diet to Non-Mediterranean Countries. What Is and What Is Not the Mediterranean Diet. Nutrients.

[69] Godfray H.C.J., Aveyard P., Garnett T., Hall J.W., Key T.J., Lorimer J., Pierrehumbert R.T., Scarborough P., Springmann M., Jebb S.A. (2018). Meat Consumption, Health, and the Environment. Science.

[70] Dixon K.A., Michelsen M.K., Carpenter C.L. (2023). Modern Diets and the Health of Our Planet: An Investigation into the Environmental Impacts of Food Choices. Nutrients.

[71] Vilarnau C., Stracker D.M., Funtikov A., da Silva R., Estruch R., Bach-Faig A. (2018). Worldwide Adherence to Mediterranean Diet between 1960 and 2011. Eur. J. Clin. Nutr..

[72] Saulle R., Semyonov L., La Torre G. (2013). Cost and Cost-Effectiveness of the Mediterranean Diet: Results of a Systematic Review. Nutrients.

[73] Page M.J., McKenzie J.E., Bossuyt P.M., Boutron I., Hoffmann T.C., Mulrow C.D., Shamseer L., Tetzlaff J.M., Akl E.A., Brennan S.E. (2021). The PRISMA 2020 statement: An updated guideline for reporting systematic reviews. BMJ.

