# Supplementary material for: Mediterranean Diet, Obesity-Related Metabolic Cardiovascular Disorders, and Environmental Sustainability: A Systematic Review
Source: Nutrients. 2025 Jun 15;17(12):2005. doi: 10.3390/nu17122005 (PMC12195730; doi:10.3390/nu17122005)
Supplement: Supplementary file 1 [file nutrients-17-02005-s001.zip › nutrients-3662414-supplementary.pdf]

PRISMA 2020 Checklist

Table S1

Section and Topic	Item #	Checklist item	Location where item is reported
**TITLE**			Lines
Title	1	Identify the report as a systematic review.	3
**ABSTRACT**			Lines
Abstract	2	See the PRISMA 2020 for Abstracts checklist.	18
**INTRODUCTION**			Lines
Rationale	3	Describe the rationale for the review in the context of existing knowledge.	95
Objectives	4	Provide an explicit statement of the objective(s) or question(s) the review addresses.	97-99
**METHODS**			Lines
Eligibility criteria	5	Specify the inclusion and exclusion criteria for the review and how studies were grouped for the syntheses.	127-134
Information sources	6	Specify all databases, registers, websites, organisations, reference lists and other sources searched or consulted to identify studies. Specify the date when each source was last searched or consulted.	111
Search strategy	7	Present the full search strategies for all databases, registers and websites, including any filters and limits used.	Table 1
Selection process	8	Specify the methods used to decide whether a study met the inclusion criteria of the review, including how many reviewers screened each record and each report retrieved, whether they worked independently, and if applicable, details of automation tools used in the process.	140-147
Data collection process	9	Specify the methods used to collect data from reports, including how many reviewers collected data from each report, whether they worked independently, any processes for obtaining or confirming data from study investigators, and if applicable, details of automation tools used in the process.	161-162
Data items	10a	List and define all outcomes for which data were sought. Specify whether all results that were compatible with each outcome domain in each study were sought (e.g. for all measures, time points, analyses), and if not, the methods used to decide which results to collect.	150-153
	10b	List and define all other variables for which data were sought (e.g. participant and intervention characteristics, funding sources). Describe any assumptions made about any missing or unclear information.	154-160
Study risk of bias assessment	11	Specify the methods used to assess risk of bias in the included studies, including details of the tool(s) used, how many reviewers assessed each study and whether they worked independently, and if applicable, details of automation tools used in the process.	164-166
Effect measures	12	Specify for each outcome the effect measure(s) (e.g. risk ratio, mean difference) used in the synthesis or presentation of results.	-
Synthesis methods	13a	Describe the processes used to decide which studies were eligible for each synthesis (e.g. tabulating the study intervention characteristics and comparing against the planned groups for each synthesis (item #5)).	-
	13b	Describe any methods required to prepare the data for presentation or synthesis, such as handling of missing summary statistics, or data conversions.	-
	13c	Describe any methods used to tabulate or visually display results of individual studies and syntheses.	201 & 205
	13d	Describe any methods used to synthesize results and provide a rationale for the choice(s). If meta-analysis was performed, describe the model(s), method(s) to identify the presence and extent of statistical heterogeneity, and software package(s) used.	-
	13e	Describe any methods used to explore possible causes of heterogeneity among study results (e.g. subgroup analysis, meta-regression).	-
	13f	Describe any sensitivity analyses conducted to assess robustness of the synthesized results.	-
Reporting bias assessment	14	Describe any methods used to assess risk of bias due to missing results in a synthesis (arising from reporting biases).	-

PRISMA

PRISMA 2020 Checklist

Section and Topic	Item #	Checklist item	Location where item is reported
Certainty assessment	15	Describe any methods used to assess certainty (or confidence) in the body of evidence for an outcome.	-
**RESULTS**			Lines
Study selection	16a	Describe the results of the search and selection process, from the number of records identified in the search to the number of studies included in the review, ideally using a flow diagram.	Figure 1
	16b	Cite studies that might appear to meet the inclusion criteria, but which were excluded, and explain why they were excluded.	179-186
Study characteristics	17	Cite each included study and present its characteristics.	Table 2
Risk of bias in studies	18	Present assessments of risk of bias for each included study.	Table S2
Results of individual studies	19	For all outcomes, present, for each study: (a) summary statistics for each group (where appropriate) and (b) an effect estimate and its precision (e.g. confidence/credible interval), ideally using structured tables or plots.	288-333
Results of syntheses	20a	For each synthesis, briefly summarise the characteristics and risk of bias among contributing studies.	Table 2
	20b	Present results of all statistical syntheses conducted. If meta-analysis was done, present for each the summary estimate and its precision (e.g. confidence/credible interval) and measures of statistical heterogeneity. If comparing groups, describe the direction of the effect.	-
	20c	Present results of all investigations of possible causes of heterogeneity among study results.	208-286
	20d	Present results of all sensitivity analyses conducted to assess the robustness of the synthesized results.	-
Reporting biases	21	Present assessments of risk of bias due to missing results (arising from reporting biases) for each synthesis assessed.	-
Certainty of evidence	22	Present assessments of certainty (or confidence) in the body of evidence for each outcome assessed.	-
**DISCUSSION**			Lines
Discussion	23a	Provide a general interpretation of the results in the context of other evidence.	335-438
	23b	Discuss any limitations of the evidence included in the review.	439-463
	23c	Discuss any limitations of the review processes used.	439-463
	23d	Discuss implications of the results for practice, policy, and future research.	408-411
**OTHER INFORMATION**			Lines
Registration and protocol	24a	Provide registration information for the review, including register name and registration number, or state that the review was not registered.	107
	24b	Indicate where the review protocol can be accessed, or state that a protocol was not prepared.	106
	24c	Describe and explain any amendments to information provided at registration or in the protocol.	-
Support	25	Describe sources of financial or non-financial support for the review, and the role of the funders or sponsors in the review.	494
Competing interests	26	Declare any competing interests of review authors.	497
Availability of data, code and other materials	27	Report which of the following are publicly available and where they can be found: template data collection forms; data extracted from included studies; data used for all analyses; analytic code; any other materials used in the review.	495-496

PRISMA 2020 Checklist

From: Page MJ, McKenzie JE, Bossuyt PM, Boutron I, Hoffmann TC, Mulrow CD, et al. The PRISMA 2020 statement: an updated guideline for reporting systematic reviews. BMJ 2021;372:n71. doi: 10.1136/bmj.n71.
This work is licensed under CC BY 4.0. To view a copy of this license, visit https://creativecommons.org/licenses/by/4.0/

Table S2

Cross sectional studies

	Article					
Question	Gualtieri et al., 2022	Llanaj & Hanley-Cook, 2021	Kocaadam-Bozkurt & Bozkurt, 2023	García, Pastor, et al., 2023	Pınarlı Falakacılar & Yücecan, 2024	Teixeira et al., 2024
1. Were the criteria for inclusion in the sample clearly defined?	Not applicable	Yes	Yes	Yes	Yes	Yes
2. Were the study subjects and the setting described in detail?	Yes	Yes	Yes	Yes	Yes	Yes
3. Was the exposure measured in a valid and reliable way?	Yes	Yes	Yes	Yes	Yes	Yes
4. Were objective, standard criteria used for measurement of the condition?	Yes	Yes	Yes	Yes	Yes	Yes
5. Were confounding factors identified?	Yes	No	No	Yes	No	No
6. Were strategies to deal with confounding factors stated?	No	No	No	Yes	No	No
7. Were the outcomes measured in a valid and reliable way?	Yes	Yes	Yes	Yes	Yes	Yes
8. Was appropriate statistical analysis used?	Yes	Yes	Yes	Yes	Yes	Yes

Cohort studies

	Article			
Question	Fresán et al., 2019	Tan & Shin, 2023	Koelman et al., 2023	Maritano et al., 2024
1. Were the two groups similar and recruited from the same population?	Yes	Yes	Yes	Yes
2. Were the exposures measured similarly to assign people to both exposed and unexposed groups?	Yes	Yes	Yes	Yes
3. Was the exposure measured in a valid and reliable way?	Yes	Yes	Yes	Yes
4. Were confounding factors identified?	Yes	Yes	Yes	Yes
5. Were strategies to deal with confounding factors stated?	Yes	Yes	Yes	Yes
6. Were the groups/participants free of the outcome at the start of the study (or at the moment of exposure)?	No	No	No	No
7. Were the outcomes measured in a valid and reliable way?	Yes	Yes	Yes	Yes
8. Was the follow up time reported and sufficient to be long enough for outcomes to occur?	Yes	Unclear	Yes	Yes
9. Was follow up complete, and if not, were the reasons to loss to follow up described and explored?	Yes	Unclear	Yes	Yes
10. Were strategies to address incomplete follow up utilized?	Yes	Yes	Yes	Yes
11. Was appropriate statistical analysis used?	Yes	Yes	Yes	Yes

Randomized controlled trial

Question		Article
Internal Validity	Choice - Comments/Justification	Monserrat-Mesquida et al., 2023
Bias related to selection and allocation		
1. Was true randomization used for assignment of participants to treatment groups?	The randomization procedure was internet-based and blinded to all staff and to the principal investigators of each centre.	Yes
2. Was allocation to treatment groups concealed?	Computer-generated random allocation was centrally elaborated in blocks of six subjects and stratified by sex, age (<65, 65–70, >70) and centre	Yes
3. Were treatment groups similar at the baseline?	Both groups were balanced	Yes
Bias related to administration of intervention/exposure		
4. Were participants blind to treatment assignment?	It was not possible	No
5. Were those delivering the treatment blind to treatment assignment?	They were not the caregivers	Yes
6. Were treatment groups treated identically other than the intervention of interest?		No
Bias related to assessment, detection and measurement of the outcome		
7. Were outcome assessors blind to treatment assignment?	They were not the caregivers	
Outcome 1	BMI	Yes
Outcome 2	Glucose, glycosylated hemoglobin (HbA1c),Glucose, glycosylated hemoglobin (HbA1c),triglycerides (TG), total cholesterol, high-density lipoprotein cholesterol (HDL-cholesterol),low-density lipoprotein cholesterol (LDL-cholesterol), aspartate aminotransferase (AST),alanine aminotransferase (ALT), and γ-glutamyl transferase (GGT) were measured usingstandardized clinical procedures	Yes
Outcome 3	DII	Yes
8. Were outcomes measured in the same way for treatment groups?		
Outcome 1	BMI	Yes
Outcome 2	Glucose, glycosylated hemoglobin (HbA1c),Glucose, glycosylated hemoglobin (HbA1c),triglycerides (TG), total cholesterol, high-density lipoprotein cholesterol (HDL-cholesterol),low-density lipoprotein cholesterol (LDL-cholesterol), aspartate aminotransferase (AST),alanine aminotransferase (ALT), and γ-glutamyl transferase (GGT) were measured usingstandardized clinical procedures	Yes
Outcome 3	DII	Yes
9. Were outcomes measured in a reliable way		
Outcome 1	BMI	Yes
Outcome 2	Glucose, glycosylated hemoglobin (HbA1c),Glucose, glycosylated hemoglobin (HbA1c),triglycerides (TG), total cholesterol, high-density lipoprotein cholesterol (HDL-cholesterol),low-density lipoprotein cholesterol (LDL-cholesterol), aspartate aminotransferase (AST),alanine aminotransferase (ALT), and γ-glutamyl transferase (GGT) were measured usingstandardized clinical procedures	Yes
Outcome 3	DII	Yes
Bias related to participant retention		
10. Was follow up complete and if not, were differences between groups in terms of their follow up adequately described and analysed?		
Outcome 1	Overweight	
Result 1	BMI	Unclear
Outcome 2	Inflammation	
Result 1	DII	Unclear
Outcome 3	Blood parameters	
Result 1	Glucose, glycosylated hemoglobin (HbA1c),triglycerides (TG), total cholesterol, high-density lipoprotein cholesterol (HDL-cholesterol),low-density lipoprotein cholesterol (LDL-cholesterol), aspartate aminotransferase (AST),alanine aminotransferase (ALT), and γ-glutamyl transferase (GGT) were measured usingstandardized clinical procedures	Unclear
Statistical Conclusion Validity		
11. Were participants analysed in the groups to which they were randomized?		
Outcome 1	Overweight	
Result 1	BMI	No
Outcome 2	Inflammation	
Result 1	DII	No
Outcome 3	Blood parameters	
Result 1	Glucose, glycosylated hemoglobin (HbA1c),triglycerides (TG), total cholesterol, high-density lipoprotein cholesterol (HDL-cholesterol),low-density lipoprotein cholesterol (LDL-cholesterol), aspartate aminotransferase (AST),alanine aminotransferase (ALT), and γ-glutamyl transferase (GGT) were measured usingstandardized clinical procedures	No
12. Was appropriate statistical analysis used?		
Outcome 1	Overweight	
Result 1	BMI	Yes
Outcome 2	Inflammation	
Result 1	DII	Yes
Outcome 3	Blood parameters	
Result 1	Glucose, glycosylated hemoglobin (HbA1c),triglycerides (TG), total cholesterol, high-density lipoprotein cholesterol (HDL-cholesterol),low-density lipoprotein cholesterol (LDL-cholesterol), aspartate aminotransferase (AST),alanine aminotransferase (ALT), and γ-glutamyl transferase (GGT) were measured usingstandardized clinical procedures	Yes
13. Was the trial design appropriate and any deviations from the standard RCT design (individual randomization, parallel groups) accounted for in the conduct and analysis of the trial?		Yes
